# Serum levels of superoxide dismutases in patients with benign paroxysmal positional vertigo

**DOI:** 10.1042/BSR20193917

**Published:** 2020-05-20

**Authors:** Jing Li, Rui Wu, Bin Xia, Xinhua Wang, Mengzhou Xue

**Affiliations:** 1 Department of Rehabilitation, The Second Affiliated Hospital of Zhengzhou University, Zhengzhou 45000, China; 2 Department of Neurology, The Fifth Affiliated Hospital of Zhengzhou University, Zhengzhou 45000, China

**Keywords:** Benign paroxysmal positional vertigo, oxidative stress, recurrence, superoxide dismutases

## Abstract

**Objective**: To investigate the possible role of superoxide dismutases (SODs) in the development of benign paroxysmal positional vertigo (BPPV) and recurrence events in a 1-year follow-up study.

**Methods**: This was a prospective one-center study. A total of 204 patients with BPPV and 120 age-and sex matched healthy subjects were included. The levels of SOD between patients and control cases were compared. The levels of SOD between posterior semicircular canal (PSC) and horizontal semicircular canal (HSC) were also compared. In the 1-year follow-up, recurrence events were confirmed. The influence of SOD levels on BPPV and recurrent BPPV were performed by binary logistic regression analysis.

**Results**: The serum levels of SOD in patients with BPPV were lower than in those control cases (*P*<0.001). Levels of SOD did not differ in patients with PSC and HSC (*P*=0.42). As a categorical variable, for per interquartile range (IQR) increment of serum level of SOD, the unadjusted and adjusted risks of BPPV would be decreased by 72% (with the odds ratio [OR] of 0.28 [95% confidence interval (CI): 0.21–0.37], *P*<0.001) and 43% (0.57 [0.42–0.69], *P*<0.001), respectively. Recurrent attacks of BPPV were reported in 50 patients (24.5%). Patients with recurrent BPPV had lower levels of SOD than in patients without (*P*<0.001). For per IQR increment of serum level of SOD, the unadjusted and adjusted risks of BPPV would be decreased by 51% (with the OR of 0.49 [95% CI: 0.36–0.68], *P*<0.001) and 24% (0.76 [0.60–0.83], *P*<0.001), respectively.

**Conclusion**: Reduced serum levels of SOD were associated with higher risk of BPPV and BPPV recurrence events.

## Introduction

Oxidative stress as a concept in redox biology and medicine had been formulated in 1985; at the beginning of 2015, approximately 138000 PubMed entries show for this term [1]. Oxidative stress is two-sided: whereas excessive oxidant challenge causes damage to biomolecules, maintenance of a physiological level of oxidant challenge, termed as oxidative eustress, is essential for governing life processes through redox signaling [2]. Oxidative stress can cause cellular dysfunction, severe tissue injury and play role in microvascular injury [3,4]. Recent studies have shown that oxidative stress was related to cardiovascular diseases [5], stroke [6] and their risk factors, such as diabetes [7], hypertension [8], metabolic syndrome and obesity [9].

In otolaryngology, the association between oxidative stress and laryngeal cancer [10], hearing loss [11], otitis media [12], chronic tonsillitis [13], rhinosinusitis [14] and other conditions has been proposed. A previous study found that the occurrence of inflammatory reaction and oxidative stress may cause abnormal lipid metabolism in the body and promote the occurrence of vascular vertigo, and platelet activation may be involved in its formation [15].

Benign paroxysmal positional vertigo (BPPV) is the most frequent peripheral vestibular disorder and is particularly seen among older patients suffering from vertigo [16]. BPPV includes two types: posterior semicircular canal (PSC) and horizontal semicircular canal (HSC) [17], and canalithiasis of the PSC might be caused in at least 85% of patients [18]. In a recent study, it has been revealed that calcium metabolism and its relationship with oxidative stress might play a role in the development of BPPV [19], while another study suggested a role of oxidative stress in the development of BPPV, through both calcium metabolism and the direct toxic effects of free oxygen radicals, including the triggering of apoptosis [17].

Superoxide dismutases (SODs) are antioxidant proteins that convert superoxide into hydrogen peroxide [20]. There are three distinct members of this metalloenzyme family in mammals: SOD1 (Cu/ZnSOD), SOD2 (MnSOD) and SOD3 (ecSOD) [21]. SODs are increasingly recognized for their regulatory functions in growth, metabolism and oxidative stress responses, which are also crucial for cancer development and survival [21]. Therefore, we hypothesized that SOD might play a role in the pathophysiology of BPPV. The present study, thus, aimed to investigate the possible role of SOD in the development of BPPV and BPPV recurrence events in a 1-year follow-up study in Chinese patients.

## Methods

### Patients

From May 2017 to May 2018, 214 patients with BPPV admitted to the Department of Neurology, The Second Affiliated Hospital of Zhengzhou University, Zhengzhou, China were included. For the diagnosis of BPPV, the Dix–Hallpike maneuver was applied to patients with vestibular complaints such as vertigo and imbalance. Torsional nystagmus with latency, fatigability and lasting shorter than 60 s in the Dix–Hallpike maneuver were considered to indicate BPPV [22]. Patients with: (1) hearing loss; (2) a history of otologic surgery; (3) neurologic disorders and autoimmune disorders; (4) malignancy; (5) cardiovascular disease, hypertension, endocrine disorders including diabetes mellitus and hypothyroidism, infectious diseases or other inflammatory conditions; (6) other factors, such as a history of head trauma, vestibular neuritis, Meniere’s disease, migraines, ear surgery or sudden hearing loss, having a hip or lumbar spine fracture were excluded.

One hundred and twenty age- and sex-matched healthy subjects without vertigo complaints or chronic illnesses from Health Care Centre of our hospital were included.

### Controls cases and clinical data

For each included case, age, sex, body mass index (BMI), blood pressure (systolic blood pressure [SBP] and diastolic blood pressure [DBP]), regular physical activity habits, smoking, drinking and family history of BPPV were recorded. Intensity of BPPV was assessed by the patients and expressed as visual analog scale (VAS) score (0–10, 0 indicated no vertigo and 10 indicated severe attacks of vertigo) [23].

All included subjects received a complete physical and neurotological examination. All the patients were evaluated within 2 weeks from the symptom onset. A typical history of brief attacks of positional vertigo was obtained from all patients with BPPV in whom the apparent etiology was absent and described as idiopathic [23]. The two types of BPPV (PSC and HSC) were diagnosed. The additional characteristics of a short-latency, limited-duration intensity characterized by crescendo and decrescendo elements were also noted in conjunction with this pattern of nystagmus of intense vertigo [24]. PSC BPPV was treated by the Epley’s maneuver, whereas HSC BPPV was treated by the Barbecue maneuver, and these maneuvers had to be repeated in ten cases [25]. In the 1-year follow-up, we defined recurrence of BPPV as that BPPV recurred more than 1 month after successful reposition, which led to the identification of nystagmus with video Frenzel glasses and consequently to the diagnosis of BPPV [26].

### Laboratory testing

Peripheral serum samples were collected from the BPPV group at the admission (during vertigo attack) and at the first month after the successful treatment with positioning maneuvers in patients with BPPV. Serum samples from healthy volunteers were collected during routine medical check-up. The samples were centrifuged for 10 min at 1500×***g***, after which the serum was separated and stored at −80°C until further analysis. Repeated freeze–thaw cycles were avoided to prevent loss of bioactive substances. Serum level of SOD was measured by colorimetry according to the manufacturer’s instructions. The coefficients of variation (CVs) of inter-assay and intra-assay for the samples containing 80, 160 and 240 U/ml of SOD were 5.0–8.0 and 6.0–9.0%, respectively. The lower detection limit was 10 U/ml and the line range was 10–250 U/ml. In addition, the serum level of C-reactive protein (CRP) and homocystinuria (HCY) were also tested by standard laboratory method.

### Statistical analysis

Percentages for categorical variables and medians (interquartile ranges, IQRs) for continuous variables were used for expressing the results. Univariate data on demographic and clinical features were compared by Mann–Whitney U-test (continuous data) or Chi-Square test (categorical data) as appropriate. Correlations among continuous variables were assessed by the Spearman rank-correlation coefficient. The influence of SOD levels on BPPV and recurrent BPPV were performed by binary logistic regression analysis, which allows adjustment for possible confounding factors (age, sex, BMI, SBP, DBP, smoking, drinking, VAS score, regular exercise habit, different semicircular canals and CRP/HCY). Results were expressed as adjusted odds ratios (ORs) with the corresponding 95% confidence interval (CI). Receiver operating characteristic (ROC) curves with area under the curve (AUC) were utilized to evaluate the accuracy of serum SOD to predict BPPV and recurrent BPPV [27]. The cut-off value of SOD was defined according to ROC curves (point at which the sum of sensitivity and specificity is maximum), and patients were divided into two groups (reduced and normal). All statistical analyses were performed with SPSS for Windows, version 22.0 (SPSS Inc., Chicago, IL, U.S.A.) and GraphPad Prism 5.0. Statistical significance was defined as *P*<0.05.

## Results

### Basic results

In the present study, 241 patients with BPPV were screened and analyzed, and 204 patients were included (2 with hearing loss; 1 with a history of otologic surgery; 6 with neurologic disorders and autoimmune disorders; 2 with malignancy; 11 with cardiovascular disease, hypertension, endocrine disorders including diabetes mellitus and hypothyroidism; 5 with infectious diseases or other inflammatory conditions; 1 with a history of head trauma; 1 with vestibular neuritis; 1 with Meniere’s disease; 5 without clinical information and 2 patients who refused to participate were excluded). The median age was 38 (IQR: 32–44) years, 50.5% were men and 59.5% were women. In those patients, 81.4% (166) were diagnosed with PSC and 18.6% (38) were with HSC. At admission, the median VAS score was 3 points (IQR: 1–4). Descriptive characteristics of patients with BPPV and control cases are presented in the Table 1. As shown in the Table 1, no significant difference of variables, such as age, sex, BMI, SBP, DBP, smoking, drinking, regular exercise habit and family history of BPPV were found between the two groups (*P*>0.05, all). Furthermore, the laboratory results suggested that serum levels of CRP and HCY were higher in BPPV than in control cases (*P*<0.05, all).

**Table 1:** Clinical characteristics of patients and controls^*^

	BPPV	Controls	*P*^†^
*n*	204	120	
Age, years	38 (32–44)	38 (31–43)	0.57
Sex: male	103 (50.5)	60 (50.0)	0.93
BMI, kg/m^2^	25.3 (24.1–27.0)	24.5 (24.3–27.5)	0.18
SBP, mmHg	108 (99–127)	110 (97–125)	0.72
DBP, mmHg	77 (70–84)	70 (68–83)	0.52
Smoking habit	42 (20.6)	21 (17.5)	0.50
Drinking habit	29 (14.2)	15 (12.5)	0.66
Family history of BPPV	15 (7.4)	6 (5.0)	0.41
Regular exercise habit	24 (11.8)	17 (14.2)	0.53
VAS score	3 (1–4)	—	
Semicircular canals		—	
PSC	166 (81.4)		
HSC	38 (18.6)		
Laboratory testing, median (IQR)			
SOD, U/ml	136.5 (121.0–159.0)	173.0 (164.5–188.7)	<0.001
HCY, μmol/l	12.4 (10.0–17.8)	11.3 (9.7–16.4)	0.012
CRP, mg/l	4.8 (3.2–7.2)	4.3 (2.8–5.9)	0.006

* Percentages for categorical variables and medians (IQRs) for continuous variables were used for expressing the results.

† The *P*-value was tested by Mann–Whitney U-test or Chi-Square test.

### Characteristics of BPPV

Serum levels of SOD did not differ statistically in patients with PSC and HSC (*P*=0.42). In patients with PSC, the right side was affected in 108 patients (65.1%), while the left side in 58 patients (34.9%). Serum SOD levels also did not differ statistically in those two groups (*P*=0.18). As a continuous variable, a negative correlation between VAS score and SOD was found (r [Spearman] = −0.225; *P*=0.001). Serum SOD levels were associated with age (r = −0.164, *P*=0.019), HCY (r = −0.303, *P*<0.001) and CRP (r = −0.218, *P*=0.002), while were not associated with other factors, such as sex, BMI, SBP, DBP, smoking, drinking, regular exercise habit and family history of BPPV (*P*>0.05, all). The Spearman coefficients found were all low and, thus, weak.

### Serum SOD levels and risk of BPPV

**Figure 1:**
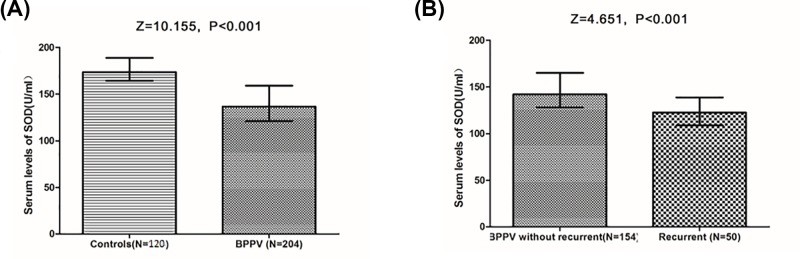
Serum levels of SOD in different groups (**A**) Serum levels of SOD in patients with BPPV and control cases. (**B**) Serum levels of SOD in patients with and without recurrent BPPV in the 1-year follow-up. Mann–Whitney U-test. All data are medians and IQRs.

As shown in Figure 1A, the serum levels of SOD in patients with BPPV were lower than in those control cases (136.5 [IQR: 121.0–159.0] vs. 173.0 [164.5–188.7] U/ml; *P*<0.001). To identify the predict value of SOD for BPPV, univariate and multiple binary logistic regression analyses were performed. As a continuous variable, for each 1 U/ml increase in serum level of SOD, the unadjusted and adjusted risk of BPPV would be decreased by 5% (with the OR of 0.95 [95% CI: 0.94–0.96], *P*<0.001) and 3% (0.97 [0.95–0.99], *P*<0.001), respectively. Multiple logistic regression analyses adjusted for age, sex, BMI, SBP, DBP, smoking, drinking, regular exercise habit, family history of BPPV, serum levels of CRP and HCY. As a categorical variable, for per IQR increment of serum level of SOD, the unadjusted and adjusted risk of BPPV would be decreased by 72% (with the OR of 0.28 [95% CI: 0.21–0.37], *P*<0.001) and 43% (0.57 [0.42–0.69], *P*<0.001), respectively.

**Figure 2:**
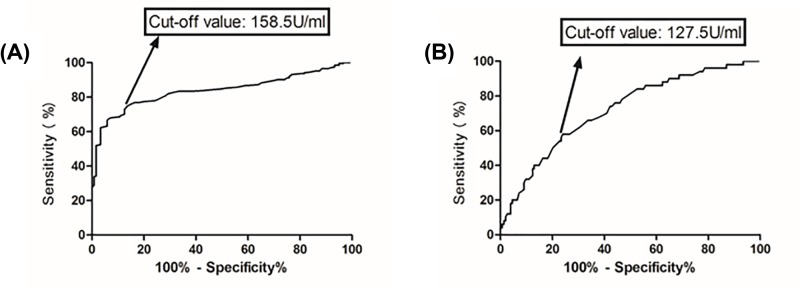
Receiver operator characteristic curve demonstrating sensitivity as a function of 1-specificity for predicting the BPPV or recurrent BPPV based on serum level of SOD (**A**) Receiver operator characteristic curve demonstrating sensitivity as a function of 1-specificity for predicting the BPPV based on serum level of SOD. (**B**) Receiver operator characteristic curve demonstrating sensitivity as a function of 1-specificity for predicting the recurrent BPPV based on serum level of SOD.

Based on the ROC curve, the optimal cut-off value of serum level of SOD as an indicator for diagnosis of BPPV was projected to be 158.5 U/ml, which yielded a sensitivity of 74.5% and a specificity of 86.7%, with the AUC at 0.83 (95% CI: 0.74–0.90), Figure 2A. Furthermore, according to this cut-off value, the patients were divided into two groups (normal vs. reduced). In multivariate analysis, there was a decreased risk of BPPV associated with normal SOD (OR: 0.72, 95% CI: 0.61–0.85; *P*<0.001) after adjusting for possible confounders.

### Serum levels SOD and risk of recurrent BPPV

Recurrent attacks of BPPV were reported in 50 patients (24.5%; 95% CI: 18.7–30.4%) in the 1-year follow-up. The median time of recurrence was 5 months (IQR: 3–7). As shown in the Figure 1B, patients with recurrent BPPV had lower levels of SOD than those patients without recurrence events (122.5 [IQR: 108.8–138.8] U/ml vs. 142.0 [128.5–165.0] U/ml; *P*<0.001). To identify the predict value of SOD for recurrent BPPV, univariate and multiple binary logistic regression analyses were performed. As a continuous variable, for each 1 U/ml increase in serum level of SOD, the unadjusted and adjusted risk of BPPV would be decreased by 3% (with the OR of 0.97 [95% CI: 0.95–0.98], *P*<0.001) and 2% (0.98 [0.94–0.99], *P*<0.001), respectively. Multiple logistic regression analyses adjusted for age, sex, BMI, SBP, DBP, smoking, drinking, VAS score, regular exercise habit, family history of BPPV, different semicircular canals, serum levels of CRP and HCY. As a categorical variable, for per IQR increment of serum level of SOD, the unadjusted and adjusted risk of BPPV would be decreased by 51% (with the OR of 0.49 [95% CI: 0.36–0.68], *P*<0.001) and 24% (0.76 [0.60–0.83], *P*<0.001), respectively.

Based on the ROC curve, the optimal cut-off value of serum level of SOD as an indicator for diagnosis of recurrent BPPV was projected to be 127.5 U/ml, which yielded a sensitivity of 58.0% and a specificity of 76.0%, with the AUC at 0.72 (95% CI: 0.64–0.80), Figure 2B. Furthermore, according to this cut-off value, the patients were divided into two groups (normal vs. reduced). In multivariate analysis, there was a decreased risk of recurrent BPPV associated with normal SOD (OR: 0.85, 95% CI: 0.73–0.94; *P*<0.001) after adjusting for possible confounders.

### Subgroup analysis

**Figure 3:**
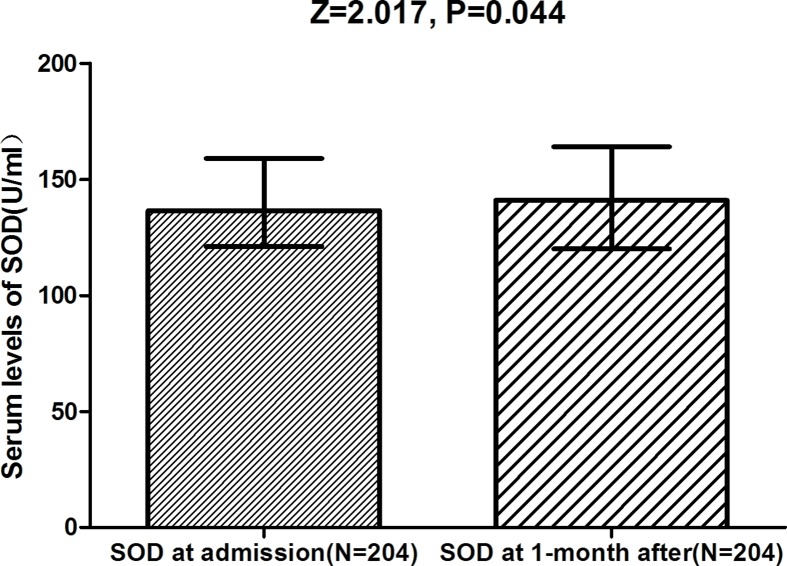
Serum levels of SOD in BPPV patients at admission and at the first month after the successful treatment Mann–Whitney U-test. All data are medians and IQRs.

There was a significant elevated in serum SOD level 1 month after the vertigo attack compared with its values during the attack (141.0 [120.0–164.0] vs*.* 136.5 [IQR: 121.0–159.0] U/ml; *P*=0.044; Figure 3), while this level is still lower than control cases (*P*<0.001).

## Discussion

The occurrence of inflammatory reaction and oxidative stress may play role in the occurrence of vascular vertigo and BPPV [15,17]. Several prior studies have explored the association between serum oxidative stress markers’ concentrations and BPPV [19,28]. To the best of our knowledge, it is the first time to measure serum level of SOD in Chinese patients with BPPV and further assess its association with BPPV recurrence events. The main findings were as following: (1) reduced levels of SOD were associated with increased risk of BPPV; (2) reduced levels of SOD were associated with increased risk of BPPV recurrence events and might be useful in identifying recurrent BPPV at risk for early prevention strategies; (3) effective treatment can increase serum levels of SOD. These findings are beneficial to the treatment of patients and provide new ideas for clinical diagnosis and treatment.

Consistent with our findings, Güçlütürk et al. [19] confirmed the role of oxidative stress, using native thiol/sulfide (SH/SS) homeostasis as a novel indicator, in the etiology of BPPV. Recent studies have suggested that oxidative stress and inner ear diseases are related. Brosel et al. [29] reported a strong link between oxidative stress, the related apoptosis of cochlear cells and age-related hearing loss. Tsai et al. [28] reported increased levels of oxidative stress markers in blood samples from patients with BPPV. Goto et al. [30] studied the probable role of angiitis and diacron reactive oxygen metabolites (d-ROMs) in BPPV; they found higher levels of vascular cell adhesion molecule-1 and d-ROM in BPPV with long-lasting vertigo attacks. Another study found that the total antioxidant status was lower in the BPPV group and there was no significant difference between the BPPV and the control groups regarding total oxidant status and the oxidative stress index [31].

In the present study, we found that there was significant difference in serum levels of SOD in the patients with BPPV before treatment vs. after treatment. However, one study found that the increase in oxidative stress did not respond to the treatments that were administered to patients with BPPV [17], and another study also showed that the levels of the antioxidant paraoxonase in patients with BPPV before and after treatment did not have obvious difference [19]. More further studies should be carried out to assess the role of treatment on the levels of oxidative stress.

The pathophysiological mechanisms by which specific plasma levels of SOD play role in the BPPV are not fully understood. Unfortunately, this cross-sectional design prevented us from inferring any cause–effect relationship of SOD with BPPV. However, clinical studies indicated that SOD may cause rather than be a consequence of BPPV. First, Talaat et al. [32] demonstrated that BPPV may be associated with low bone mineral density and vitamin D deficiency. Uberti et al. [33] reported that vitamin D protects human endothelial cells from oxidative stress through the autophagic and survival pathways. Second, calcium metabolism and oxidative stress are closely linked. The endoplasmic reticulum, the major organelle for calcium storage, has the capability to increase the influx of calcium under stress conditions, which in turn triggers the cascade reaction of ROS formation in mitochondria [34]. Both oxidative stress and calcium influx into the mitochondria cause rupture of the mitochondrial outer membrane and apoptosis under reperfusion injury [35]. When all these studies are considered together, BPPV may have an oxidative stress-based pathogenesis [19]. At last, oxidative stress and inflammation are closely related pathophysiological processes, one of which can be easily induced by another [36]. SOD might play role in the BPPV through inflammation reaction [19]. In this study, we also found an association between CRP and SOD.

The present study included limitations. First, we did not have access to the inner ear in order to uncover the actual chemistry of the endolymph in real time. Second, it is clearly unrealistic to homogenize all environmental and demographic factors for all enrolled subjects. Third, it was a single-center study and all the patients were Chinese. In addition, the present study was conducted on a small patient group. Fourth, further studies with larger samples are needed to evaluate and compare other oxidative stress markers, such as paraoxonase and arylesterase, as well as the total antioxidant status in patients with BPPV. Fifth, the accuracy of the diagnostic tests and therapeutic maneuvers should be considered. Future protocols should include the use of VNG and reposition devices, since these provide pinpoint accuracy in diagnosis and treatment of BPPV [37]. At last, observational study design did not allow drawing any causal relationship. There may be an association between SOD and effective treatment, but it is not necessarily causative.

## Conclusion

Reduced serum levels of SOD were associated with higher risk of BPPV and BPPV recurrence events, requiring further efforts to clarify the exact mechanism for early prevention strategies. Further studies are proposed to confirm this association.

## Data Availability

Please contact corresponding author for data requests.

## Abbreviations

AUC: area under the curve
BMI: body mass index
BPPV: benign paroxysmal positional vertigo
CI: confidence interval
CRP: C-reactive protein
DBP: diastolic blood pressure
d-ROM: diacron reactive oxygen metabolite
HCY: homocystinuria
HSC: horizontal semicircular canal
IQR: interquartile range
OR: odds ratio
PSC: posterior semicircular canal
ROC: receiver operating characteristic
SBP: systolic blood pressure
SOD: superoxide dismutase
VAS: visual analog scale
